# Comprehensive evaluation of microRNA-10b in digestive system cancers reveals prognostic implication and signaling pathways associated with tumor progression

**DOI:** 10.7150/jca.51303

**Published:** 2021-05-13

**Authors:** Yi Shen, Xiaolei Dai, Haibo Chen, Shuwei Zhai, Qiliang Peng, Shang Cai, Yaqun Zhu, Jian Huan, Yuntian Shen

**Affiliations:** 1 Department of Radiation Oncology, The Affiliated Suzhou Science & Technology Town Hospital of Nanjing Medical University, Suzhou, China.; 2 Department of General Surgery, Suzhou TCM Hospital Affiliated to Nanjing University of Chinese Medicine.; 3 Department of Radiotherapy & Oncology, The Second Affiliated Hospital of Soochow University, Suzhou, China.; 4 Institute of Radiotherapy & Oncology, Soochow University, Suzhou, China.

**Keywords:** digestive system cancers, microRNA-10b, prognosis prediction, biomarker

## Abstract

**Background:** Digestive system cancers (DSCs) have been recognized to be linked with high morbidity and mortality. Recent studies have reported that microRNA-10b (miR-10b) is abnormally expressed in DSCs and associated with prognosis. However, the inconclusive results and unknown underlying mechanisms promoted us to perform this study.

**Methods:** We systematic searched several databases for eligible studies and conducted quantitative analysis for evidence regarding the associations between miR-10b and survival outcome of DSCs. We also performed a series of bioinformatics analyses to uncover the potential mechanisms.

**Results:** A total of 32 eligible studies with 3392 patients were included. Increased miR-10b expression was linked with unfavorable overall survival (OS) in DSCs (HR=1.72; 95% CI: 1.30-2.27; P <0.001). When stratified by tumor type, the impact of miR-10b overexpression on poor prognosis was observed in colorectal cancer, gastric cancer, hepatocellular carcinoma, and esophageal carcinoma, but not in pancreatic cancer. Subsequently, we predicted the targets of miR-10b and conducted functional enrichment analyses. The results disclosed that miR-10b targets were predominantly enriched in some vital biological terms and pivotal signaling pathways associated with tumor progression including cell cycle, FoxO, proteoglycans, central carbon metabolism, p53, Notch, HIF-1, focal adhesion, AMPK, and pancreatic cancer. Moreover, a protein-protein interaction (PPI) network was also constructed to identify the top ten hub genes and significant modules and demonstrated the underlying interactions among them.

**Conclusion:** Our results indicated that miR-10b could act as a significant biomarker in the prognosis DSCs. However, more research should be performed to test these findings.

## Introduction

Digestive system cancers (DSCs) have become a major public health threat, including esophageal squamous cell carcinoma, gastric cancer, colorectal cancer, hepatocellular carcinoma, gallbladder cancer and pancreatic cancer 1. Despite significant advancements in the diagnosis and therapy strategies for DSCs in recent decades, these types of diseases remain as leading causes of morbidity and mortality of patients across the world 2. In recent years, considerable efforts were made to identify prognostic factors for improving risk stratification and personalized treatment in DSCs. Nevertheless, as the complexity and heterogeneity of cancers, DSCs patients at same stages of disease could response to the same treatment differently and lead to different clinical outcomes 3. Thus, it is necessary to explore novel reliable prognostic biomarkers with high sensitivity and specificity that provide effective therapeutic strategies to enhance life quality and survival outcomes of patients with DSCs.

MicroRNAs, about 22 nucleotides, are a member of highly conserved and single-stranded non-coding RNAs, which function to be mRNA level regulators by regulating messenger RNA translation and degradation 4. In recent years, deregulated microRNAs have been identified in multiple types of human diseases and cancers 5. Moreover, a series of studies have already demonstrated that microRNAs also have a significant effect on cancer progression and may be used as promising biomarkers for cancer diagnosis, prognosis and therapy effects 6. Thus, more and more researches concentrate on the microRNAs as the promising biomarkers of DSCs 7,8.

Among these well-studied microRNAs, microRNA-10b (miR-10b) may be one of the most important members. There are several studies indicating that miR-10b high expression is highly related to clinical outcome of patients with various types of malignant tumors, especially in breast cancer 9,10. Recently, miR-10b has been identified as a reliable prognostic indicator in DSCs, with a promising role in predicting the survival outcomes for these patients 11. Although accumulating evidence suggests that miR-10b is aberrantly expressed in DSCs, there has been no clear consensus about its role regarding the prognostic value in such diseases. To date, it remains unclear why miR-10b may be associated with the survival outcome of patients with DSCs.

Therefore, we performed this study to evaluate the prognostic significance of miR-10b expression in DSCs by analyzing all the available data coming from a comprehensive search. Furthermore, based on above results, we applied several bioinformatics analysis methods for an integrated characterization of miR-10b regarding the potential mechanism associated with tumor progression.

## Material and methods

### Data sources and searches

To enroll all associated articles assessing the prognostic value of miR-10b overexpression in digestive system cancer, we searched PubMed Central, Web of Science, and EMBASE (up to December 12, 2020) with the following search terms: (microRNA-10b OR miR-10b OR miRNA-10b) and (esophageal OR gastric OR stomach OR hepatocellular OR pancreatic OR rectal OR rectum OR colon OR colorectal OR gastrointestinal OR digestive) and (cancer OR carcinoma OR tumor OR neoplasm OR malignancy). All related studies from the lists of references of retrieved articles were manually analyzed to obtain other potentially relevant reports.

### Eligibility criteria

Studies were included if (i) they studied the patients with digestive system cancers which were diagnosed with pathology; (ii) they explored the associations between miR-10b expression levels and prognosis; (iii) They directly reported hazard risks (HRs) of overall survival (OS) and the 95% confidence intervals (CIs) or provided information to estimate HRs; (iv) they were published in English.

Studies were excluded if (i) they were not associated with our aim; (ii) they were published as meta-analysis, case reports, conference abstracts, reviews or letters; (iii) there was insufficient data available in the study; and (iv) if they were written in languages other than English.

### Data extraction and document assessment

The extracted data included information on first author, publication year, study population, patient age, sample size, cancer type, cancer stage, detection method, follow-up period, end point, and risk estimates (HRs and 95% CIs). If the studies only provided Kaplan-Meier survival curves, then data about the HRs with 95% CIs was extracted and estimated by the method illustrated by Tierney 12. Afterwards, we employed the Newcastle-Ottawa Scale (NOS) for observational studies to evaluate the quality of them 13. The above parts of this study were performed by two authors independently. When disagreement occurred between the two reviewers, a third investigator was invited to provide help for meeting the consensus.

### Data synthesis and analysis

The relationship of miR-10b with OS was exhibited as a weighted average of study-specific estimates of the HR and 95% CI. Heterogeneity between publications was estimated using Q statistics and Higgins I^2^ statistic. If statistical heterogeneity was significant (I^2^>50% or P<0.05), the random-effects model was used for quantitative synthesis; if not, the fixed-effects model would be adopted 14. When heterogeneity was significant, we applied subgroup, meta-regression and sensitivity analyses 15. We evaluated publication bias using Begg's funnel plot and Egger's regression asymmetry test 16. STATA (version 12.0, College Station, TX) was applied for all statistical calculations and analysis. For all analyses, a 2-sided P-value of <0.05 was considered as statistically significant.

### Identification of the regulated genes of miR-10b

The regulated genes by miR-10b were collected from the miRTarBase database. The powerful tool covers maybe the most integratively annotated and experimentally confirmed microRNA-target interactions. The update in 2020 with more improvements has promoted the database to be more comprehensive and integrative 17.

### Functional annotation of miR-10b by KEGG and GO analysis

Gene ontology (GO) and Kyoto Encyclopedia of Genes and Genomes (KEGG) pathway analysis were applied for further understanding and simulating higher-order functional activities cells or organisms from the genomic information of miR-10b 18,19. In our study, GO analysis was performed for annotating the regulated genes of miR-10b based on three major sections containing biological processes (BP), cellular components (CC) and molecular functions (MF). In the current study, Database for Annotation, Visualization, and Integrated Discovery (DAVID) was employed for accomplishing GO functional and KEGG pathway enrichment analyses 20. Both GO and KEGG terms with a P-value < 0.05 were considered as statistically significant.

### Construction of the PPI network and integrative analysis

Search Tool for the Retrieval of Interacting Genes (STRING) is an online database developed for investigating the protein-protein interaction (PPI) data 21. To evaluate the potential associations among the regulated genes of miR-10b, we uploaded them into STRING and collected the interactions with the combined score of >0.4 for constructing the PPI network 22. CytoNCA plug-in of Cytoscape software was employed to identify the crucial hub proteins while MCODE plug-in in Cytoscape was employed to screen the significant network modules 23. Ultimately, the module nodes were enriched for functional KEGG pathways. P<0.05 was considered to reveal significance.

### Validation of hub genes

First, we performed the pathway enrichment analysis of the identified hub genes. Then, a literature search regarding the associations among the pathways and DSCs in PubMed was carried out. Finally, the Gene Expression Profiling Interactive Analysis (GEPIA) database was used to further verify the prognostic value of the hub genes in DSCs 24.

## Results

### Identification of eligible studies

**Figure 1:**
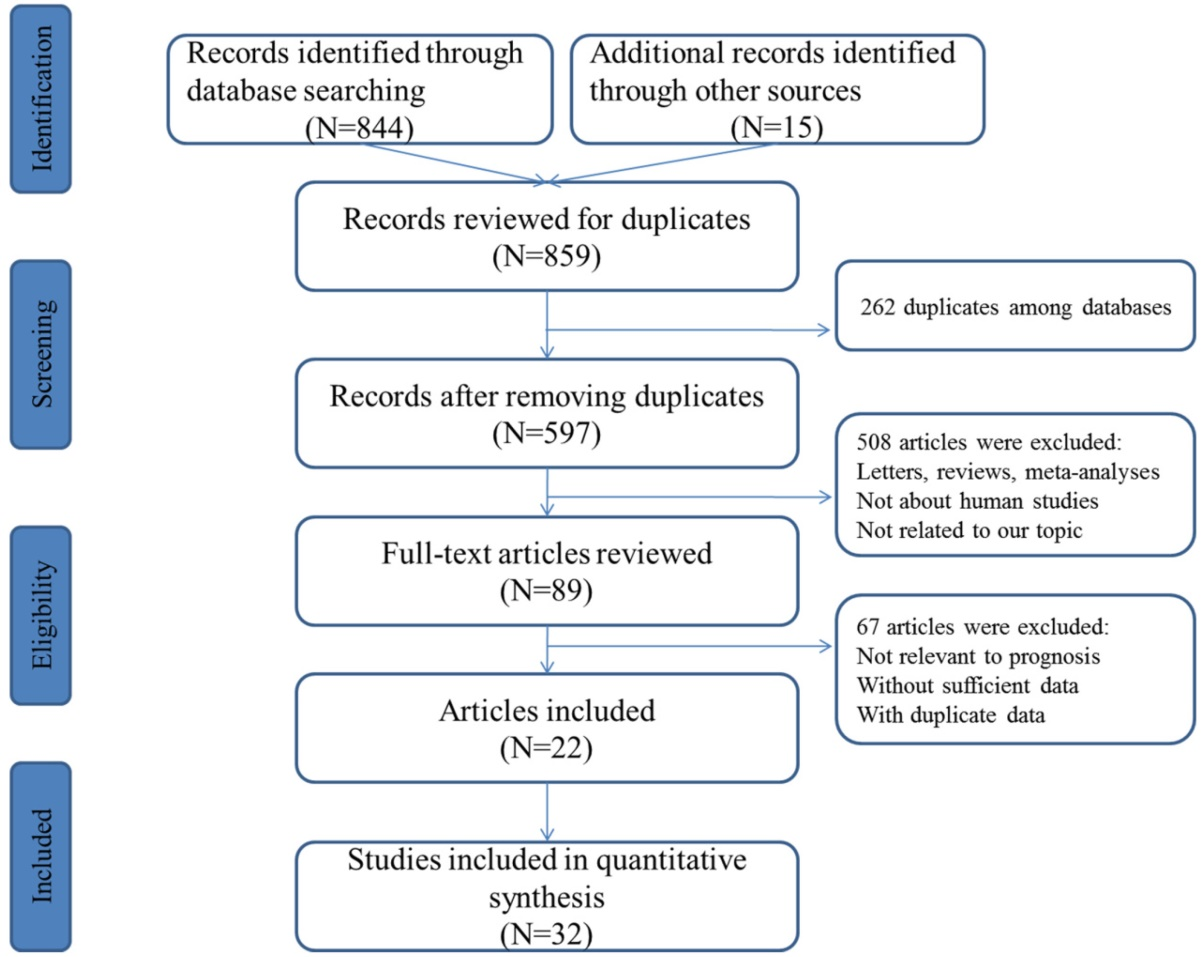
Flowchart presenting the steps of literature search and selection.

The concise process of literature selection was illustrated at **Figure 1**. A total of 492 publications were retrieved through searching the selected databases. After the elimination of duplicates and carefully screening the titles, abstracts and full-texts of these publications, subsequently, 22 articles including 32 studies were enrolled for quantitative analysis 25-46.

### Basic characteristics of the selected studies

Study characteristics of the eligible studies were summarized at **Table 1**. All of the eligible studies were published from 2011 to 2020. Among the studies included, nine studies focused on colorectal cancer, ten concentrated on gastric cancer, four were about hepatocellular cancer, seven were about pancreatic cancer and two were about esophageal squamous cell carcinoma. Studies concerning Asians occupied the largest proportion of patient populations among all primary literatures (n=19), followed by Europeans (n=7), Americans (n=5), and Africans (n=1). The majority of the included studies focused on tissue miR-10b. Most of the enrolled studies had a prospective design and applied qRT-PCR for the detection of miR-10b expression levels. According to the NOS criteria, the study quality score varied from 5 to 7, which suggested that the study quality was moderate to high.

### Correlation between miR-10b expression and OS

**Figure 2:**
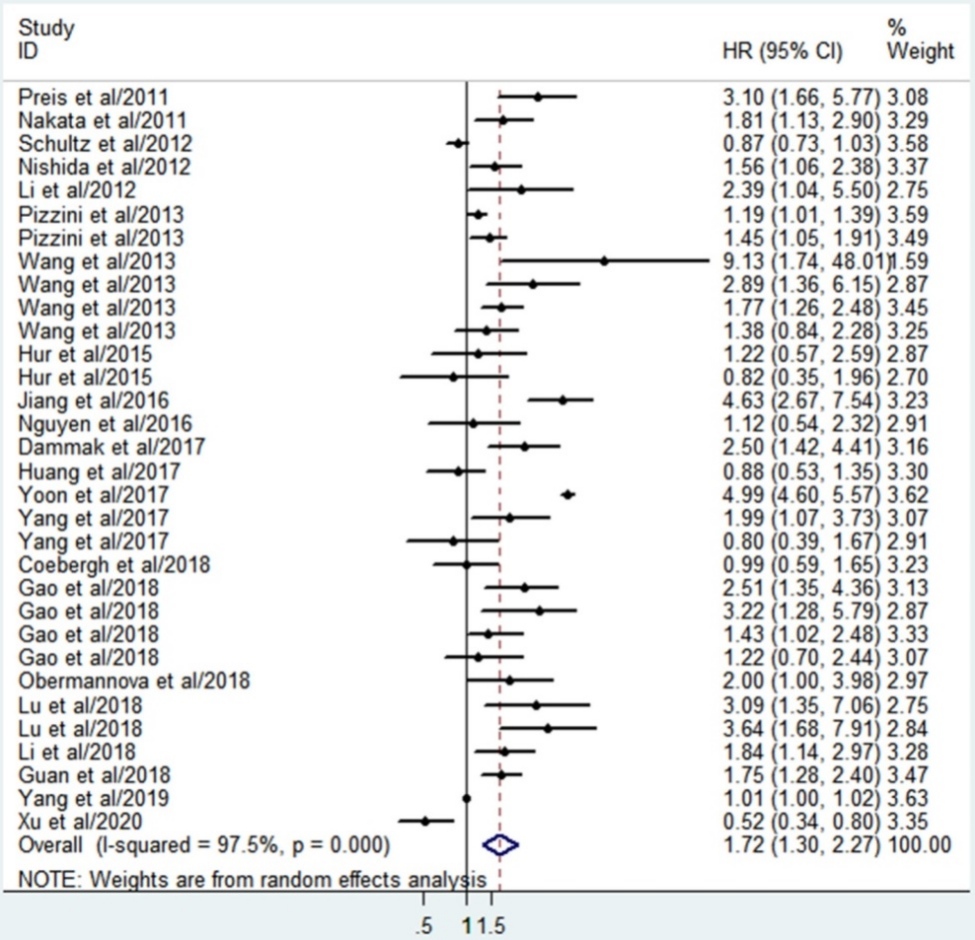
Forest plot for the association between miR-10b expression and the overall survival of patients with digestive system cancers.

A total of 32 studies involving 3392 patients described the associations between miR-10b and OS. Since significant heterogeneity among the studies was observed (I^2^=97.5%, P<0.001), the randomized effects model was conducted to calculate the pooled HR and 95%CI. The pooled analysis results revealed that elevated miR-10b was significantly connected with poor OS of DSCs patients (HR=1.72; 95% CI: 1.30-2.27; P <0.001), which indicated miR-10b may serve as a promising prognostic biomarker for DSCs (**Figure 2**).

### Heterogeneity analysis

Due to the heterogeneity, subgroup analysis was conducted by the ethnicity, sample sizes, and cancer types (**Table 2**). A significant correlation between miR-10b overexpression and a poor OS was demonstrated in patients with colorectal cancer (HR=1.45, 95% CI: 1.13-1.85, P=0.003), gastric cancer (HR=1.76, 95% CI: 1.33-2.32, P<0.001), hepatocellular cancer (HR=2.53, 95% CI: 1.25-5.15, P=0.01) and esophageal squamous cell carcinoma (HR=3.37, 95% CI: 1.92-5.93, P<0.001), indicating the expression level of miR-10b could be applied to predict the OS of various kinds of DSCs. However, the significant association was not observed in pancreatic cancer (HR=1.21, 95% CI: 0.79-1.86, P=0.38). In addition, the subgroup analysis results revealed that a high miR-10b may act as a predictor for worse OS in Asians (HR=1.99, 95% CI: 1.36-2.91, P<0.001), but not in Americans (HR=1.48, 95% CI: 0.95-2.31, P=0.086), and Europeans (HR=1.19, 95% CI: 0.95-1.49, P=0.134). In the analysis stratified by size of sample, miR-10b was revealed to be significantly related OS of patients in studies with sample size larger than median (HR=1.58, 95% CI: 1.24-2.01, P<0.001) and sample size fewer than median (HR=1.74, 95% CI: 1.15-2.64, P=0.009).

Furthermore, meta-regression analysis was further performed to identify the source of heterogeneity, based on the cancer type, ethnicity, and sample size. The results revealed that none of the factors might be possible source of heterogeneity.

### Sensitivity analysis and publication bias

**Figure 3:**
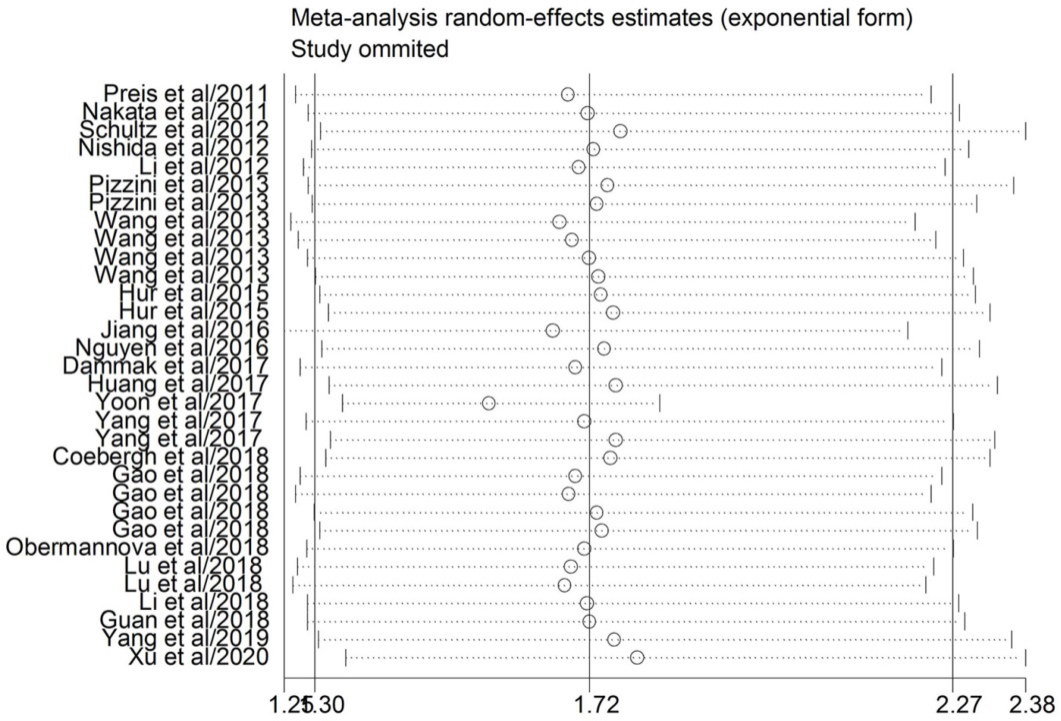
Sensitivity analysis of HR for association between miR-10b expression and overall survival.

A sensitivity analysis was carried out for evaluating the effect of a single study on the stability of the pooled results (**Figure 3**). The association continued insignificant when any single article was omitted, which indicated that the conclusions from the quantitative analysis were relatively reliable. We employed Egger's and Begg's tests to evaluate the potential publication bias in the evidence synthesis of the impact of miR-10b on OS in DSCs. The results revealed that statistically significant publication bias was observed for the overall analysis of OS in patients with DSCs (P=0.024).

### Functional and pathway enrichment analysis

**Figure 4:**
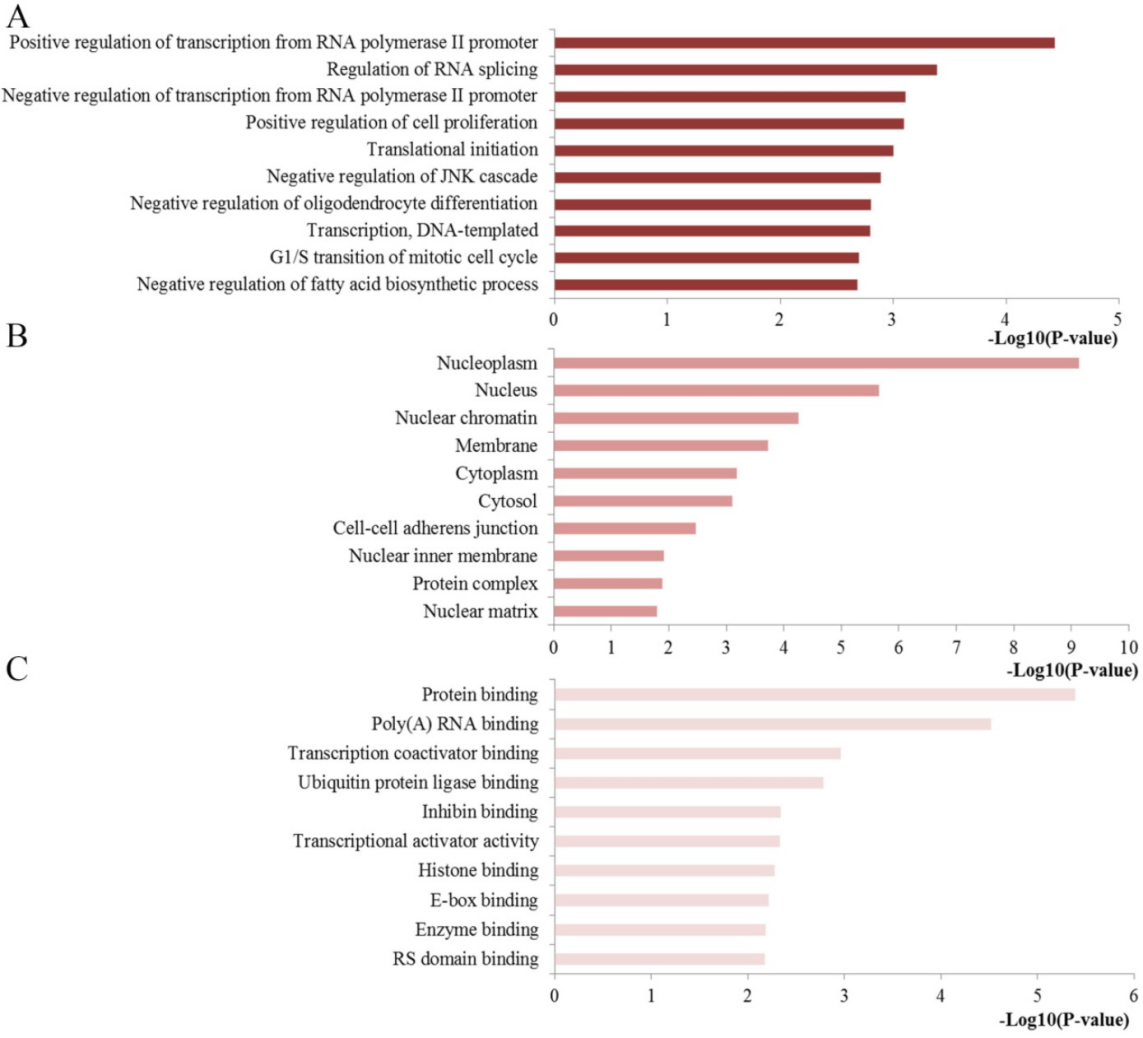
Top ten GO annotation of miR-10b regulated genes. (**A**) Biological processes; (**B**) cell component; (**C**) molecular function.

To extract biological information regarding the function and mechanisms of miR-10b, we analyzed the GO and KEGG pathway enrichment by using the online tool of DAVID. The results of the GO analysis were exhibited from three aspects: BP, CC and MF (**Figure 4**). The GO functional annotation analysis of the regulated genes by miR-10b revealed that (1) the BP section was mainly involved in regulation of transcription from RNA polymerase II promoter, regulation of RNA splicing, regulation of cell proliferation, and translational initiation; (2) the CC section was highly related to nucleoplasm, nucleus, nuclear chromatin, membrane and cytoplasm; and (3) the MF section was significantly associated with protein binding, poly(A) RNA binding, transcription coactivator binding, ubiquitin protein ligase binding and inhibin binding.

**Figure 5:**
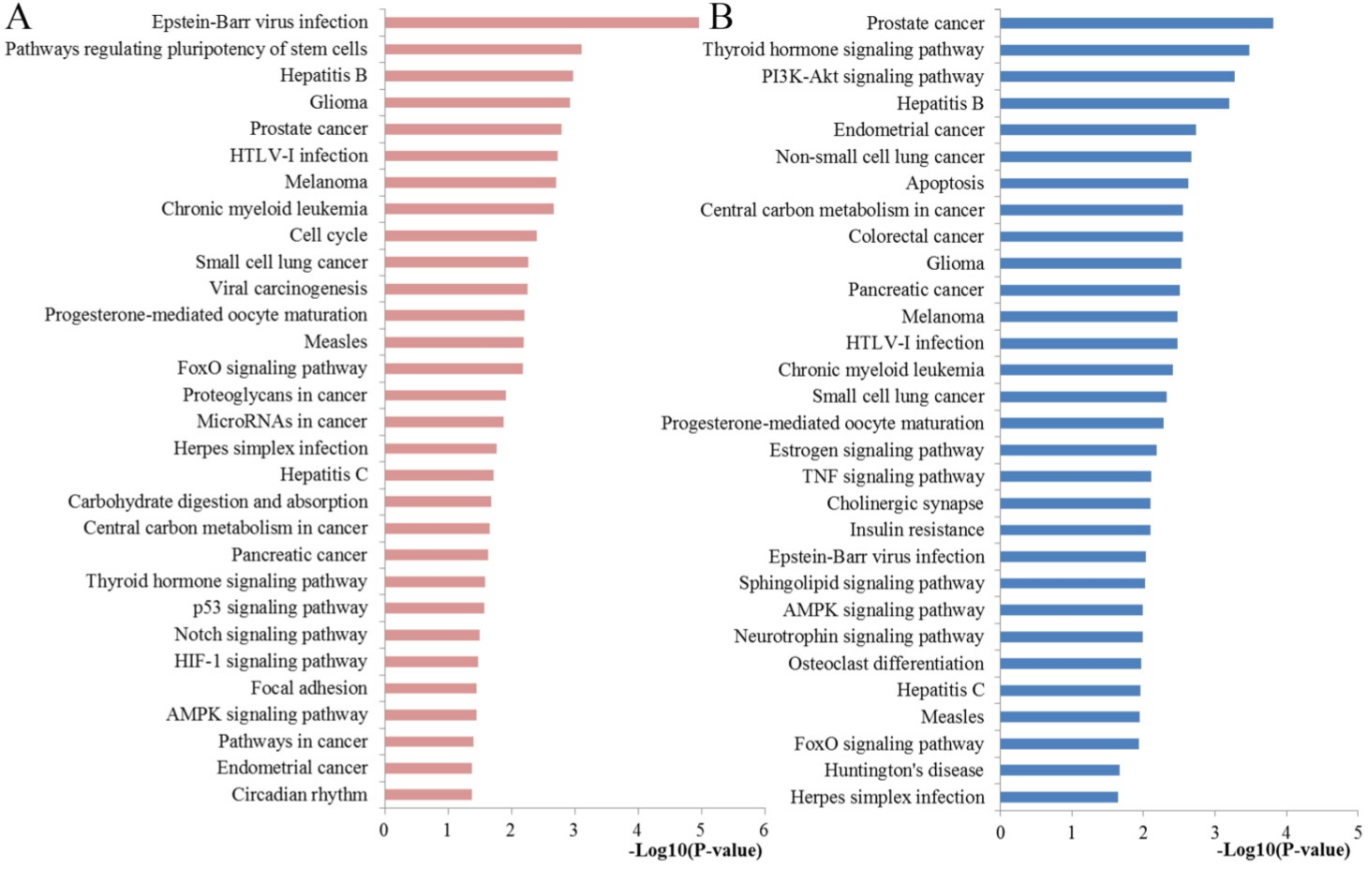
Pathway enrichment results. (**A**) Top 30 pathways enriched by all the target genes of miR-10b; (**B**) Top 30 pathways enriched by the hub nodes of miR-10b.

**Figure 6:**
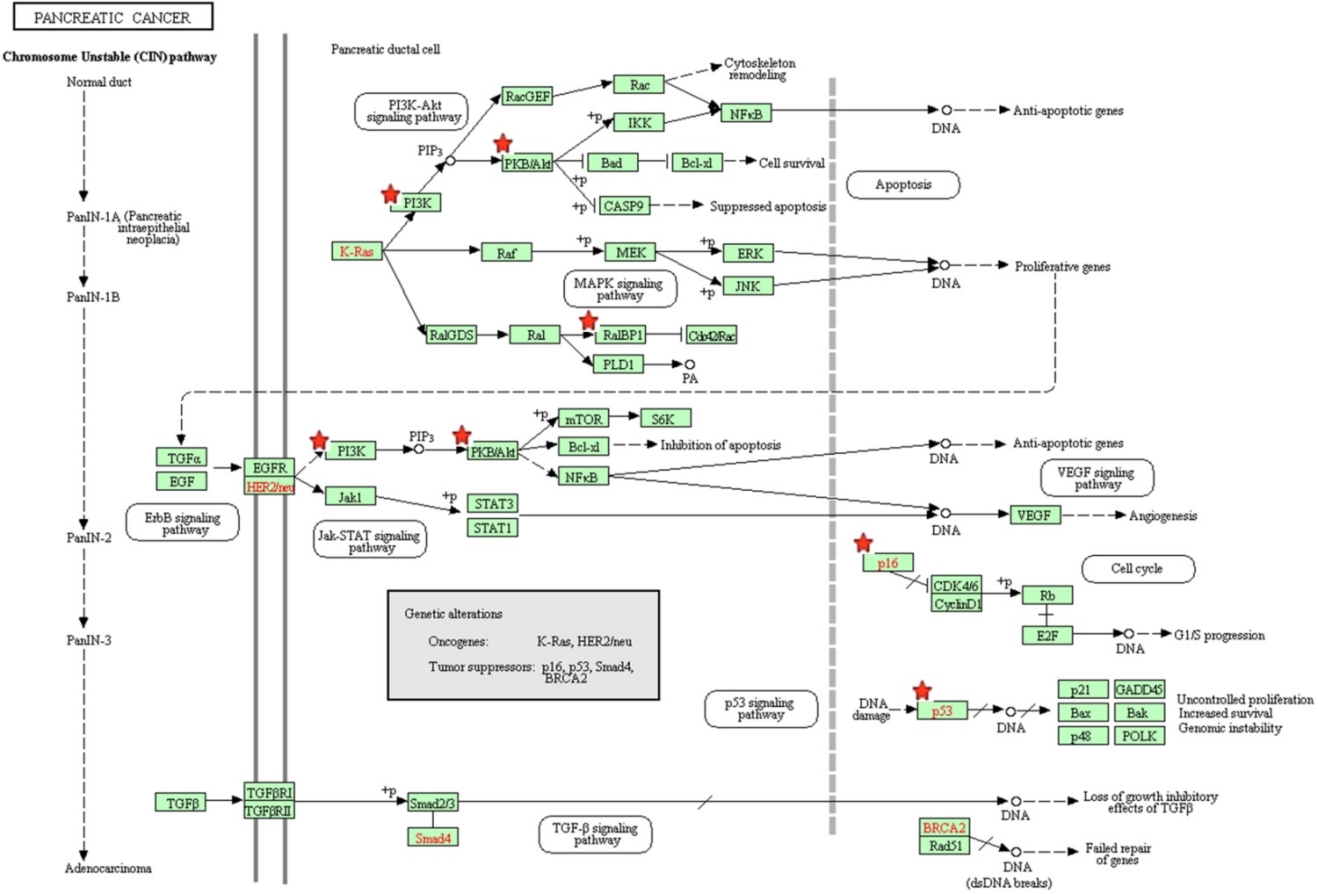
The pancreatic cancer signaling pathway enriched in KEGG. Objects with pentagrams are acting locus by mapped genes.

The results of the KEGG analysis were presented at **Figure 5**. The regulated genes by miR-10b were significantly enriched in cell cycle, FoxO signaling pathway, proteoglycans in cancer, microRNAs in cancer, central carbon metabolism in cancer, pancreatic cancer, p53 signaling pathway, Notch signaling pathway, HIF-1 signaling pathway, Focal adhesion, AMPK signaling pathway and Pathways in cancer. The pancreatic cancer pathway from KRGG was plotted at **Figure 6**, which also has close relationships with p53 signaling, cell cycle, PI3K-Akt signaling and VEGF signaling.

### Construction of the PPI network and identification of hub genes

**Figure 7:**
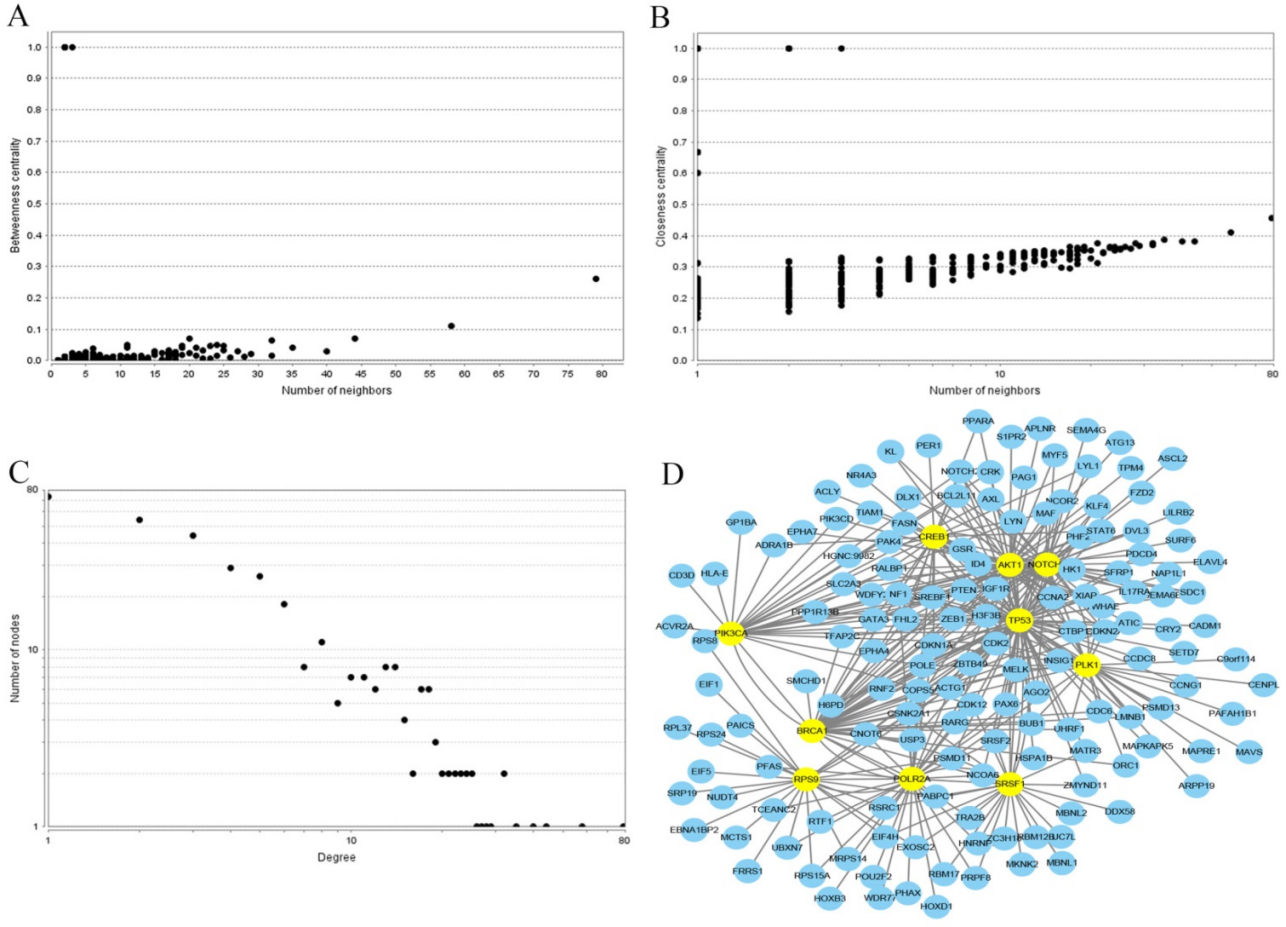
PPI network construction results. (**A**) Betweenness centrality distributions of nodes; (**B**) Closeness centrality distributions of nodes; (**C**) Degree distributions of nodes; (**D**) The sub-network reconstructed with the selected hub nodes and their first neighbor genes.

In order to investigate the correlations between the regulated genes by miR-10b, the interaction network of PPI was established using the STRING database. Each node gene in this network was subjected to statistical analysis including betweenness centrality and closeness centrality and degree centrality (**Figure 7**). Ultimately, the top ten hub nodes were identified: *AKT1*,* BRCA1*,* CREB1*,* NOTCH1*,* PIK3CA*,* PLK1*,* POLR2A*,* RPS9*,* SRSF1*, and *TP53*. The sub-network reconstructed with the selected hub nodes and their first neighbor genes was also plotted at **Figure 7**.

### Module screening and characterization of function

**Figure 8:**
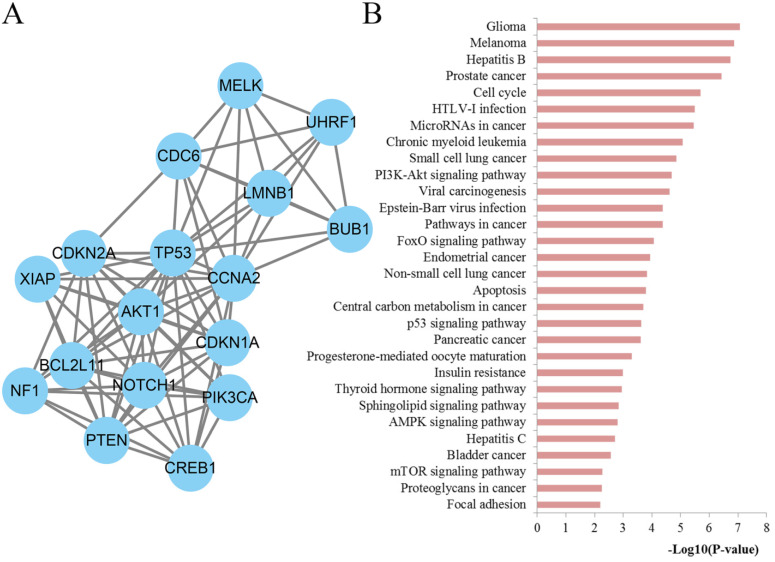
Module analysis results of the PPI network. (**A**) The most significant module in the PPI network; (**B**) Pathways enriched by all the nodes involved in the identified module.

To further explore the significance of the molecules of the DSCs associated PPI network, the module analysis was carried out by utilizing the MCODE plug-in in Cytoscape. Afterwards, KEGG pathways of those module nodes were re-analyzed via DAVID to better understand their functions (**Figure 8**). The results indicated that the module nodes were significantly enriched in cell cycle, microRNAs in cancer, PI3K-Akt signaling pathway, pathways in cancer, FoxO signaling pathway, apoptosis, central carbon metabolism in cancer, p53 signaling pathway, pancreatic cancer, sphingolipid signaling pathway, AMPK signaling pathway, mTOR signaling pathway, proteoglycans in cancer and focal adhesion.

### Functional enrichment and prognostic value assessment of hub genes

To obtain more comprehensive information about the critical pathways of the identified hub genes, KEGG pathways analysis was also conducted with DAVID tool (**Figure 5**). We found that the regulated genes by miR-10b were mainly enriched in PI3K-Akt signaling pathway, apoptosis, colorectal cancer, central carbon metabolism in cancer, pancreatic cancer, estrogen signaling pathway, TNF signaling pathway, sphingolipid signaling pathway, neurotrophin signaling pathway, AMPK signaling pathway and FoxO signaling pathway.

**Figure 9:**
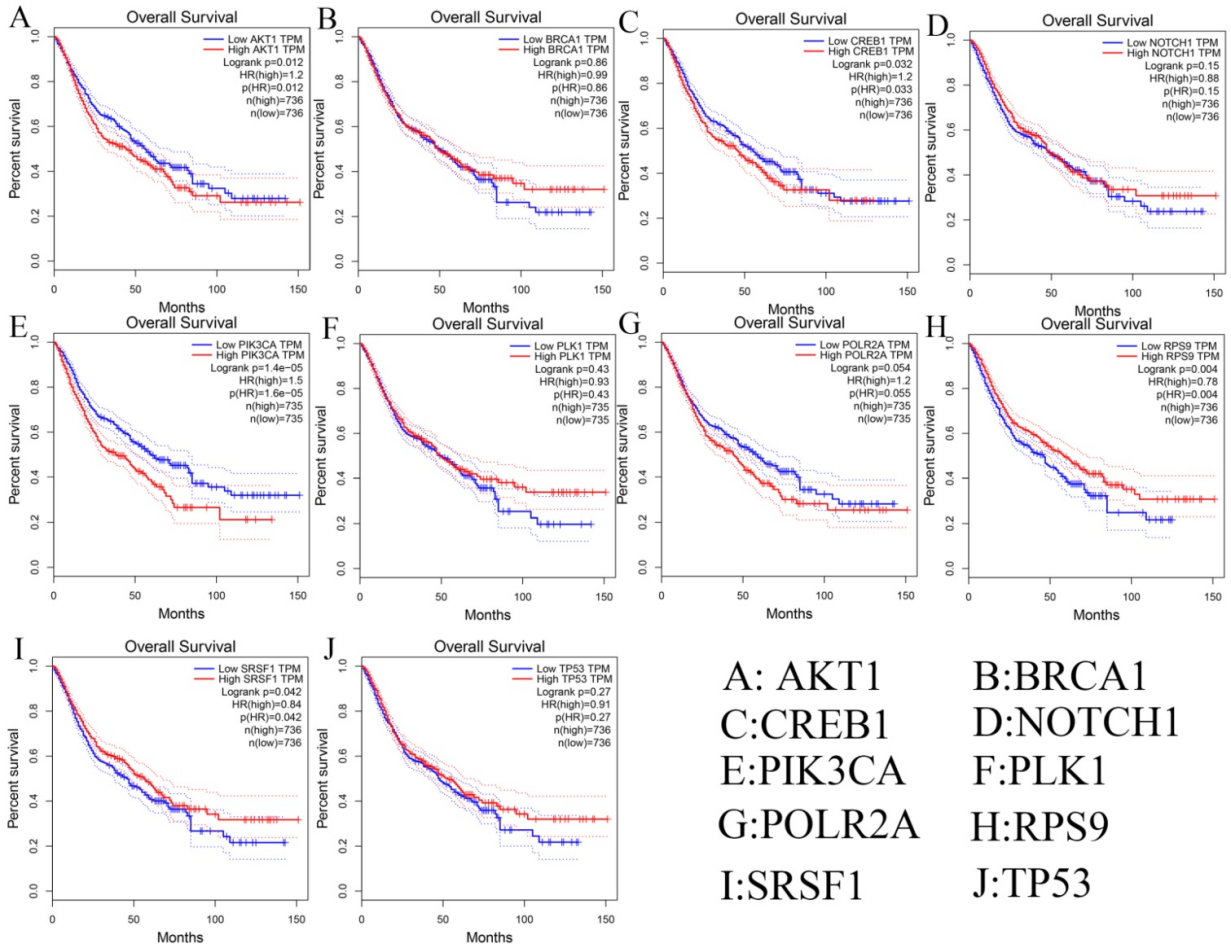
Survival analysis of ten hub genes in DSCs patients.

Besides, we investigated the prognostic values of hub genes using GEPIA database. As shown in **Figure 9**, high expression of AKT1 (logrank p=0.012), CREB1 (logrank p=0.032), and PIK3CA (logrank p=1.4e-05) predicted a poor prognosis for DSCs patients, while the high expression of RPS9 (logrank p=0.004) and SRSF1 (logrank p=0.042) predicted a favorable prognosis for DSCs patients. However, the expression levels of BRCA1 (logrank p=0.86), NOTH1 (logrank p=0.15), PLK1 (logrank p=0.43), POLR2A (logrank p=0.054), and TP53 (logrank p=0.27) were not associated with OS prognosis of DSCs patients.

## Discussion

As the early symptoms of DSCs are not obvious, the high morbidity and mortality rate of DSCs have become a major fatal problem across the world. The rapid progression and unsatisfactory survival make it inevitable to explore tumor biomarkers, which could enhance the early diagnosis and treatment effect through leading more precise and valuable information. It is now well documented that microRNAs play a pivotal role in a series of critical processes of tumorigenesis, including tumor cell mutation, proliferation, invasion, progression and metastasis. Among those microRNAs, miR-10b is of full interest to researchers for its significant role as a promising biomarker for predicting the overall survival of various malignancies. Nevertheless, to the best of our knowledge, the independent prognostic role of miR-10b in DSCs remains unclear due to different results from different studies. Therefore, the purpose of this study was to provide a comprehensive and relatively reliable conclusion about the association between miR-10b expression and prognostic value in patients diagnosed with DSCs. In addition, we also explored the potential mechanisms for miR-10b involved in the progression of DSCs by using several bioinformatics methods.

The pooled results of quantitative analysis demonstrated that high miR-10b expression was significantly associated with worse OS in DSCs (pooled HR=1.72, 95% CI: 1.30-2.27, P<0.001). Moreover, subgroup analysis by cancer type indicated that high expression of miR-10b was significantly correlated with poor OS in colorectal cancer, gastric cancer, hepatocellular cancer and esophageal squamous cell carcinoma, suggesting that miR-10b was an indicator of decreased survival rate in various types of DSCs. Then we observed that ethnicity may influence the association between miR-10b and OS in DSCs as the predictive value of miR-10b in Asians was more significant than in Americans and Europeans by subgroup analysis. Moreover, sample size seemed to have little effect on the final result as small sample size presented more significant predictive role than larger sample size. In addition, sensitivity analysis proved the reliability of the pooled results. Taken together, miR-10b may function as a promising biomarker for monitoring the progression of DSCs.

To understand whether the main biological functions of miR-10b are related to initiation and progression of DSCs, functional enrichment analysis of the regulated genes of miR-10b was performed by using the DAVID online tool. The results of GO showed that the genes regulated by miR-10b were mostly involved in some important regulation processes for the BP section, significantly linked with some crucial cell components for the CC section and highly enriched into the key molecules binding for the MF section. These enriched terms were highly associated with tumor occurrence and progression.

KEGG analysis also indicated that most of the genes regulated by miR-10b were mainly enriched in pathways including cell cycle, pathways in cancer proteoglycans in cancer, microRNAs in cancer, central carbon metabolism in cancer, pancreatic cancer, FoxO, p53, Notch, HIF-1, focal adhesion, and AMPK signaling pathway. These pathways have been well-studied and have been confirmed according to the previous experimental researches. For example, the signaling of pathways in cancer indicated that the genes regulated by miR-10b participated in a series of important pathways involved in DSCs 47. Moreover, the signaling pathway of microRNAs in cancer and pancreatic cancer demonstrated the direct association again 48. Cell cycle, perhaps the most crucial signaling in the initiation and progression of multiple types of cancers including DSCs, has been recognized as an extremely important mechanism for cell growth, development, metabolism and regeneration of eukaryotic organism 49. Proteoglycans have been identified to be crucial regulators of the cell/matrix interface and, consecutively, biological function, the discrete expression of which has been distinguished to serve a specific role during disease development in diverse cancer type including DSCs 50. It is well established that central carbon metabolism plays a crucial role in cancer cell proliferation through regulating cancer cell metabolism 51. Emerging evidence has supported the vital role of FoxO signaling in multiple cellular and physiological activities such as, cell proliferation, regulating programmed cell death, longevity, metabolism and cancer and decorating cell cycle 52. The p53 signaling, another very vital pathway, participated in regulating a plethora of biological processes ranging from development, cell signaling, and tumorigenesis to cell metabolism 53. Notch signaling pathway is highly conserved and critical in regulating various developmental processes and in the maintenance of tissue homeostasis in many cancers 54. Recent new evidence gathered so far has indicated that non-coding RNAs could not only activate or inhibit Notch signaling, but also regulate the initiation and progression of cancer through Notch signaling 55. The HIF-1 pathway has a profound effect in different types of cancer with the significant properties to induce tumor cell growth 56. Targeting HIF-1 may be adopted as a promising therapy in some kinds of cancers 57. And for the focal adhesion signaling, accumulating new evidence has revealed that it plays a vital part during the progression of tumors to a malignant phenotype 58. Last but not least, AMPK is a key factor in regulating tissue energy metabolism, controlling immune responses and modifying immunometabolism and the biological functions of immune cells 59. For a preliminary summary, the statistically significant biological processes and the KEGG pathways may present the potential mechanisms of miR-10b to some extent.

Furthermore, we analyzed the potential associations among the genes regulated by miR-10b through conducting an integrated PPI network analysis. A series of key hub genes were then identified. To explore the potential mechanisms of these hub proteins, KEGG pathway analysis was performed using DAVID online tool. The enrichment analysis indicated that these key hub proteins were enriched in apoptosis, colorectal cancer, central carbon metabolism in cancer, pancreatic cancer, PI3K-Akt, estrogen, TNF, Sphingolipid, Neurotrophin, AMPK, and FoxO signaling pathway. Most of these pathways have been reported as the most promising signaling pathway involved in the carcinogenesis of DSCs. The colorectal cancer signaling demonstrated that miR-10b was associated with DSCs 60. Apoptosis has been critically reviewed by a large amount of studies for its roles in cell growth, inflammation, differentiation, and metastasis, the abnormal activation of which may contribute to the pathogenesis of DSCs 61. There is considerable evidence that PI3K-Akt signaling plays an important role in regulating cell proliferation, growth, cell size, metabolism, and motility 62. A large body of evidence from preclinical studies reveals that estrogen has a close association with the presence of colorectal cancer initiation and progression 63. Accumulating new evidence has identified TNF to be a proinflammatory cytokine with pivotal role in coordinating tissue homeostasis through the regulation of cytokine production, cell survival, and cell death 64. Sphingolipids have been known as bioactive lipids that take part in multiple biological mechanisms, such as cell death and proliferation. Targeting sphingolipids may provide potential target structures for pharmaceutical anticancer research 65. Emerging evidence supports the critical roles of the Neurotrophin signaling as drivers of neurogenesis within the processes of development and regeneration, and they could be expressed in human cancers where they could stimulate cancer cell growth 66. These results demonstrated that these hub proteins played important roles in DSCs and could be critical therapeutic targets.

The network module might be helpful to provide novel insights into protein function explanation. Thus, we identified the significant modules through network analysis. To further investigate the biological functions of these modules, KEGG enrichment analysis was performed with the nodes involved in the selected module. The results indicated that the module nodes were mainly enriched in cell cycle, microRNAs in cancer, pathways in cancer, apoptosis, central carbon metabolism in cancer, pancreatic cancer, proteoglycans in cancer, focal adhesion, PI3K-Akt, FoxO, p53, Sphingolipid, AMPK and mTOR signaling pathway. These results were consistent with the above analysis and have been demonstrated to be an important factor for the tumorigenesis of DSCs. It is worth noting that the mTOR signaling pathway, which plays an obvious role in regulating cellular growth, proliferation, survival, metabolism, angiogenesis, and transcription 67. Targeting mTOR signaling may provide innovative therapeutic strategies for DSCs 68. The PPI network analysis will provide a new perspective on the potential mechanisms for miR-10b in DSCs. Moreover, targeting the above pathways might help develop effective strategy for the treatment of DSCs.

Recently, a number of previous published meta-analysis and review articles have studied the impact and potential mechanisms of miR-10b in DSCs. For example, Mei L et al. carried out a study to assess the prognostic and diagnostic values of miR-10b in gastric cancer based on meta-analysis and TCGA database. The results indicated that miR-10b may function as a tumor suppressor with prognostic and diagnostic values for GC, which was consistent with our expectations about the prognostic roles of miR-10b 69. Lu Y et al. studied the association between abnormal miR-10b expression and cancer risk through a meta-analysis and found that high-expression of miR-10b could be significantly related to cancer risk 70. Huang Q performed a systematic review and meta-analysis to determine the implication of miR-10b on the survival of cancer patients in 2017 and they showed that overexpression of miR-10b predicted the shorter OS for the patient with different types of cancers 71. These studies have focused on the specific prognostic value of miR-10b in DSCs and no attention in these studies has been paid to why miR-10b can act as a biomarker. In addition, Chen XL et al. found that miR-10b promoted the invasion and metastasis of human gastric cancer cells through inhibiting the expression of CSMD1, causing the activation of the NF-κB pathway while Lu YF et al. suggested up-regulated expression of miR-10b-3p caused by promoter hypomethylation contributed to the progression of esophageal squamous cell carcinoma 38,72. These studies have focused on a specific molecular mechanism of miR-10b; however, the functions of miR-10b are complex and various. Although there may be a lack of novelty, we not only conducted a comprehensive analysis of miR-10b and prognosis of DSCs, but also revealed the potential mechanisms from the overall level by using some bioinformatics analysis methods. Although not perfect, most of the findings have been successfully confirmed by recent experimental literatures. According to our findings, we would like to propose some recommendations for future research. Firstly, more attention should be paid for the impact of miR-10b on the prognosis of DSCs, especially researches beyond China and Japan. Secondly, the key genes regulated by miR-10b and the crucial pathways may provide effective strategy for the treatment of DSCs and worth further research. Last but not least, a large-scale and multicenter study will be needed to promote the clinical application of miR-10b in DSCs.

We aware that the current research may exist several significant limitations. Firstly, due to a limited size of enrolled studies of some types of cancers, the results of these types of cancers were statistically insignificant and might be less powerful. Secondly, the cut-off values in the included studies were not uniform, which might be a potential factor of heterogeneity. Moreover, potential publication bias was observed in this study as some studies that yielded negative results were generally not published. In addition, some survival data were manually extracted from Kaplan-Meier curves, which may also contribute to unavoidable bias and heterogeneity. All the eligible studies were retrospectively designed, which tended to be published when positive conclusions have been confirmed. The main findings and conclusion of our study have not been validated by biological experiment.

## Conclusions

In conclusion, the study demonstrated that high miR-10b expression level is significantly associated with poor prognosis in DSCs patients. Therefore, miR-10b may become a valuable biomarker for predicting prognosis in DSCs patients. Moreover, a series of key genes regulated by miR-10b may be involved in the initiation and progression of DSCs and ultimately affect their prognosis through some important signaling pathways. However, more clinical investigations with larger sample size should be carried out to focus on the relationship between miR-10b expression and patient prognosis.

## Figures and Tables

**Table 1:** Characteristics of the included articles

Author	Year	Country	Ethnicity	M/F	N	Age	Cancer type	TNM stage	Sample source	Methods	Endpoints	Median follow-up time	Hazard ratio
Preis et al	2011	USA	Americans	NR	95	68	Pancreatic	I-IV	Tissue	ISH	OS	NA	3.10 (1.66-5.77)
Nakata et al	2011	Japan	Asians	73/42	115	64	Pancreatic	I-IV	Tissue	RT-PCR	OS	13	1.81 (1.13-2.90)
Schultz et al	2012	Denmark	Europeans	111/114	225	64	Pancreatic	I-II	Tissue	RT-PCR	OS	24	0.87 (0.73-1.03)
Nishida et al	2012	Japan	Asians	55/33	88	65	Colorectal	I-IV	Tissue	RT-PCR	OS	48	1.56 (1.06-2.38)
Li et al	2012	China	Asians	29/5	34	50	Hepatocellular	I-IV	Tissue	RT-PCR	OS	60	2.39 (1.04-5.50)
Pizzini et al	2013	Italy	Europeans	NR	20	61	Colorectal	IV	Tissue	RT-PCR	OS	60	1.19 (1.01-1.39)
Pizzini et al	2013	Italy	Europeans	NR	26	61	Colorectal	IV	Tissue	RT-PCR	OS	60	1.45 (1.05-1.91)
Wang et al	2013	China	Asians	69/21	90	63	Gastric	I	Tissue	RT-PCR	OS	60	9.13 (1.74-48.01)
Wang et al	2013	China	Asians	80/24	104	63	Gastric	II	Tissue	RT-PCR	OS	60	2.89 (1.36-6.15)
Wang et al	2013	China	Asians	133/40	173	63	Gastric	III	Tissue	RT-PCR	OS	60	1.77 (1.26-2.48)
Wang et al	2013	China	Asians	53/19	69	63	Gastric	IV	Tissue	RT-PCR	OS	60	1.38 (0.84-2.28)
Hur et al	2015	USA	Americans	66/18	83	67	Colorectal	I-IV	Tissue	RT-PCR	OS	80	1.22 (0.57-2.59)
Hur et al	2015	Japan	Asians	102/73	175	68	Colorectal	I-IV	Tissue	RT-PCR	OS	60	0.82 (0.35-1.96)
Jiang et al	2016	China	Asians	176/70	246	60	Colorectal	I-IV	Tissue	RT-PCR	OS	60	4.63 (2.67-7.54)
Nguyen et al	2016	USA	Americans	36/19	55	48	Pancreatic	I-II	Tissue	RT-PCR	OS	34	1.12 (0.54-2.32)
Dammak et al	2017	Tunisia	Africans	NR	50	63	Colorectal	I-IV	Tissue	RT-PCR	OS	33	2.50 (1.42-4.41)
Huang et al	2017	China	Asians	134/54	188	60	Gastric	I-IV	Serum	RT-PCR	OS	>50	0.88 (0.53-1.35)
Yoon et al	2017	Korea	Asians	21/3	24	57	Hepatocellular	III-IV	Serum	RT-PCR	OS	60	4.99 (4.60-5.57)
Yang et al	2017	Germany	Europeans	NA	69	NA	Pancreatic	I-IV	Tissue	RT-PCR	OS	>60	1.99 (1.07-3.73)
Yang et al	2017	Germany	Europeans	NA	41	69	Pancreatic	I-IV	Tissue	RT-PCR	OS	>60	0.81 (0.39-1.67)
Coebergh et al	2018	Netherlands	Europeans	82/73	155	NR	Colorectal	I-II	Tissue	RT-PCR	OS	30	0.99 (0.59-1.65)
Gao et al	2018	China	Asians	13/12	25	59	Gastric	I	Tissue	ISH	OS	60	2.51 (1.35-4.36)
Gao et al	2018	China	Asians	15/14	29	59	Gastric	II	Tissue	ISH	OS	60	3.22 (1.28-5.79)
Gao et al	2018	China	Asians	25/23	48	59	Gastric	III	Tissue	ISH	OS	60	1.43 (1.02-2.48)
Gao et al	2018	China	Asians	10/8	18	59	Gastric	IV	Tissue	ISH	OS	60	1.22 (0.70-2.44)
Obermannova et al	2018	Czech	Europeans	28/39	67	68	Gastric	I-IV	Tissue	RT-PCR	OS	60	2.00 (1.00-3.98)
Lu et al	2018	China	Asians	77/16	93	60	Esophageal	I-IV	Tissue	RT-PCR	OS	80	3.09 (1.35-7.06)
Lu et al	2018	China	Asians	84/18	102	60	Esophageal	I-IV	Tissue	RT-PCR	OS	80	3.64 (1.68-7.91)
Li et al	2018	USA	Americans	NR	173	NR	Hepatocellular	I-IV	Tissue	RT-PCR	OS	60	1.84 (1.14-2.97)
Guan et al	2018	China	Asians	NR	320	NR	Hepatocellular	I-IV	Tissue	RT-PCR	OS	60	1.75 (1.28-2.40)
Yang et al	2019	USA	Americans	NR	212	68	Colorectal	I-IV	Tissue	RT-PCR	OS	60	1.01 (1.00-1.02)
Xu et al	2020	China	Asians	106/74	180	60	Pancreatic	I-IV	Tissue	RT-PCR	OS	81	0.52 (0.34-0.80)

**Abbreviation**: F, female; M, male; N, number; NR, not report; OS, overall survival.

**Table 2:** Results of subgroup and meta-regression analyses

Subgroup	Studies	HR (95%CI)	P-value	Heterogeneity (I^2^)	P_heterogeneity_	Meta-regression (*P*-value)
**Ethnicity**						P=0.182
Asians	19	1.99 (1.36-2.91)	P<0.001	93.4%	P<0.001	
Americans	5	1.48 (0.95-2.31)	P=0.086	78.6%	P=0.001	
Europeans	7	1.19 (0.95-1.49)	P=0.134	67.7%	P=0.005	
**Cancer type**						P=0.772
Colorectal	9	1.45 (1.13-1.85)	P=0.003	86.0%	P<0.001	
Gastric	10	1.76 (1.33-2.32)	P<0.001	55.9%	P=0.016	
Hepatocellular	4	2.53 (1.25-5.15)	P=0.01	94.5%	P<0.001	
Esophageal	2	3.37 (1.92-5.93)	P<0.001	0.0%	P=0.777	
Pancreatic	7	1.21 (0.79-1.86)	P=0.38	83.0%	P<0.001	
**Sample size**						P=0.550
Large (N>median)	16	1.58 (1.24-2.01)	P<0.001	87.9%	P<0.001	
Small (N< median)	16	1.74 (1.15-2.64)	P=0.002	95.4%	P=0.009	

## References

[1] Siegel RL, Miller KD, Jemal A (2019). Cancer statistics, 2019. *CA Cancer J Clin*.

[2] Bray F, Ferlay J, Soerjomataram I, Siegel RL, Torre LA, Jemal A (2018). Global cancer statistics 2018: GLOBOCAN estimates of incidence and mortality worldwide for 36 cancers in 185 countries. *CA Cancer J Clin*.

[3] Turajlic S, Sottoriva A, Graham T, Swanton C (2019). Resolving genetic heterogeneity in cancer. *Nat Rev Genet*.

[4] Treiber T, Treiber N, Meister G (2019). Regulation of microRNA biogenesis and its crosstalk with other cellular pathways. *Nat Rev Mol Cell Biol*.

[5] Matsuyama H, Suzuki HI (2019). Systems and Synthetic microRNA Biology: From Biogenesis to Disease Pathogenesis. *Int J Mol Sci*.

[6] Sun Z, Shi K, Yang S, Liu J, Zhou Q, Wang G, Song J, Li Z, Zhang Z, Yuan W (2018). Effect of exosomal miRNA on cancer biology and clinical applications. *Mol Cancer*.

[7] Sheng JQ, Liu L, Wang MR, Li PY (2018). Circular RNAs in digestive system cancer: potential biomarkers and therapeutic targets. *Am J Cancer Res*.

[8] Kawaguchi T, Komatsu S, Ichikawa D, Tsujiura M, Takeshita H, Hirajima S, Miyamae M, Okajima W, Ohashi T, Imamura T, Kiuchi J, Konishi H, Shiozaki A, Okamoto K, Otsuji E (2016). Circulating MicroRNAs: A Next-Generation Clinical Biomarker for Digestive System Cancers. *Int J Mol Sci*.

[9] Sheedy P, Medarova Z (2018). The fundamental role of miR-10b in metastatic cancer. *Am J Cancer Res*.

[10] Zhang J, Yang J, Zhang X, Xu J, Sun Y, Zhang P (2018). MicroRNA-10b expression in breast cancer and its clinical association. *PLoS One*.

[11] Zhang Y, Wang LJ, Yang HQ, Wang R, Wu HJ (2019). MicroRNA-10b expression predicts long-term survival in patients with solid tumor. *J Cell Physiol*.

[12] Wang Y, Zeng T (2013). Response to: Practical methods for incorporating summary time-to-event data into meta-analysis. *Trials*.

[13] Stang A (2010). Critical evaluation of the Newcastle-Ottawa scale for the assessment of the quality of nonrandomized studies in meta-analyses. *Eur J Epidemiol*.

[14] Higgins JP, Thompson SG, Deeks JJ, Altman DG (2003). Measuring inconsistency in meta-analyses. *BMJ*.

[15] Kriston L (2013). Dealing with clinical heterogeneity in meta-analysis. Assumptions, methods, interpretation. *Int J Methods Psychiatr Res*.

[16] Augusteijn HEM, van Aert RCM, van Assen M (2019). The effect of publication bias on the Q test and assessment of heterogeneity. *Psychol Methods*.

[17] Huang HY, Lin YC, Li J, Huang KY, Shrestha S, Hong HC, Tang Y, Chen YG, Jin CN, Yu Y, Xu JT, Li YM, Cai XX, Zhou ZY, Chen XH, Pei YY, Hu L, Su JJ, Cui SD, Wang F, Xie YY, Ding SY, Luo MF, Chou CH, Chang NW, Chen KW, Cheng YH, Wan XH, Hsu WL, Lee TY, Wei FX, Huang HD (2020). miRTarBase 2020: updates to the experimentally validated microRNA-target interaction database. *Nucleic Acids Res*.

[18] Hinderer EW 3rd, Flight RM, Dubey R, MacLeod JN, Moseley HNB (2019). Advances in gene ontology utilization improve statistical power of annotation enrichment. *PLoS One*.

[19] Kanehisa M, Goto S (2000). KEGG: kyoto encyclopedia of genes and genomes. *Nucleic Acids Res*.

[20] Dennis G Jr, Sherman BT, Hosack DA, Yang J, Gao W, Lane HC, Lempicki RA (2003). DAVID: Database for Annotation, Visualization, and Integrated Discovery. *Genome Biol*.

[21] Szklarczyk D, Gable AL, Lyon D, Junge A, Wyder S, Huerta-Cepas J, Simonovic M, Doncheva NT, Morris JH, Bork P, Jensen LJ, Mering CV (2019). STRING v11: protein-protein association networks with increased coverage, supporting functional discovery in genome-wide experimental datasets. *Nucleic Acids Res*.

[22] Otasek D, Morris JH, Boucas J, Pico AR, Demchak B (2019). Cytoscape Automation: empowering workflow-based network analysis. *Genome Biol*.

[23] Tang Y, Li M, Wang J, Pan Y, Wu FX (2015). CytoNCA: a cytoscape plugin for centrality analysis and evaluation of protein interaction networks. *Biosystems*.

[24] Tang Z, Li C, Kang B, Gao G, Li C, Zhang Z (2017). GEPIA: a web server for cancer and normal gene expression profiling and interactive analyses. *Nucleic Acids Res*.

[25] Nakata K, Ohuchida K, Mizumoto K, Kayashima T, Ikenaga N, Sakai H, Lin C, Fujita H, Otsuka T, Aishima S, Nagai E, Oda Y, Tanaka M (2011). MicroRNA-10b is overexpressed in pancreatic cancer, promotes its invasiveness, and correlates with a poor prognosis. *Surgery*.

[26] Preis M, Gardner TB, Gordon SR, Pipas JM, Mackenzie TA, Klein EE, Longnecker DS, Gutmann EJ, Sempere LF, Korc M (2011). MicroRNA-10b expression correlates with response to neoadjuvant therapy and survival in pancreatic ductal adenocarcinoma. *Clin Cancer Res*.

[27] Li QJ, Zhou L, Yang F, Wang GX, Zheng H, Wang DS, He Y, Dou KF (2012). MicroRNA-10b promotes migration and invasion through CADM1 in human hepatocellular carcinoma cells. *Tumour Biol*.

[28] Nishida N, Yamashita S, Mimori K, Sudo T, Tanaka F, Shibata K, Yamamoto H, Ishii H, Doki Y, Mori M (2012). MicroRNA-10b is a prognostic indicator in colorectal cancer and confers resistance to the chemotherapeutic agent 5-fluorouracil in colorectal cancer cells. *Ann Surg Oncol*.

[29] Schultz NA, Andersen KK, Roslind A, Willenbrock H, Wojdemann M, Johansen JS (2012). Prognostic microRNAs in cancer tissue from patients operated for pancreatic cancer-five microRNAs in a prognostic index. *World J Surg*.

[30] Pizzini S, Bisognin A, Mandruzzato S, Biasiolo M, Facciolli A, Perilli L, Rossi E, Esposito G, Rugge M, Pilati P, Mocellin S, Nitti D, Bortoluzzi S, Zanovello P (2013). Impact of microRNAs on regulatory networks and pathways in human colorectal carcinogenesis and development of metastasis. *BMC Genomics*.

[31] Wang YY, Ye ZY, Zhao ZS, Li L, Wang YX, Tao HQ, Wang HJ, He XJ (2013). Clinicopathologic significance of miR-10b expression in gastric carcinoma. *Hum Pathol*.

[32] Coebergh van den Braak RRJ, Sieuwerts AM, Lalmahomed ZS, Smid M, Wilting SM, Bril SI, Xiang S, van der Vlugt-Daane M, de Weerd V, van Galen A, Biermann K, van Krieken J, Kloosterman WP, Foekens JA, group* Ms, Martens JWM, JNM IJ (2018). Confirmation of a metastasis-specific microRNA signature in primary colon cancer. *Sci Rep*.

[33] Jiang H, Liu J, Chen Y, Ma C, Li B, Hao T (2016). Up-regulation of mir-10b predicate advanced clinicopathological features and liver metastasis in colorectal cancer. *Cancer Med*.

[34] Abdelmaksoud-Dammak R, Chamtouri N, Triki M, Saadallah-Kallel A, Ayadi W, Charfi S, Khabir A, Ayadi L, Sallemi-Boudawara T, Mokdad-Gargouri R (2017). Overexpression of miR-10b in colorectal cancer patients: Correlation with TWIST-1 and E-cadherin expression. *Tumour Biol*.

[35] Yoon EL, Yeon JE, Ko E, Lee HJ, Je JH, Yoo YJ, Kang SH, Suh SJ, Kim JH, Seo YS, Yim HJ, Byun KS (2017). An Explorative Analysis for the Role of Serum miR-10b-3p Levels in Predicting Response to Sorafenib in Patients with Advanced Hepatocellular Carcinoma. *J Korean Med Sci*.

[36] Guan L, Ji D, Liang N, Li S, Sun B (2018). Up-regulation of miR-10b-3p promotes the progression of hepatocellular carcinoma cells via targeting CMTM5. *J Cell Mol Med*.

[37] Li D, Zhang Y, Zhang H, Zhan C, Li X, Ba T, Qiu Z, E F, Lv G, Zou C, Wang C, Si L, Zou C, Li Q, Gao X (2018). CADM2, as a new target of miR-10b, promotes tumor metastasis through FAK/AKT pathway in hepatocellular carcinoma. *J Exp Clin Cancer Res*.

[38] Lu YF, Yu JR, Yang Z, Zhu GX, Gao P, Wang H, Chen SY, Zhang J, Liu MY, Niu Y, Wei XM, Wang W, Ye FJ, Zhang LX, Zhao Y, Sun GG (2018). Promoter hypomethylation mediated upregulation of MicroRNA-10b-3p targets FOXO3 to promote the progression of esophageal squamous cell carcinoma (ESCC). *J Exp Clin Cancer Res*.

[39] Obermannova R, Redova-Lojova M, Vychytilova-Faltejskova P, Grell P, Cho WC, Sachlova M, Svoboda M, Vyzula R, Slaby O (2018). Tumor Expression of miR-10b, miR-21, miR-143 and miR-145 Is Related to Clinicopathological Features of Gastric Cancer in a Central European Population. *Anticancer Res*.

[40] Yang G, Zhang Y, Yang J (2019). A Five-microRNA Signature as Prognostic Biomarker in Colorectal Cancer by Bioinformatics Analysis. *Front Oncol*.

[41] Hur K, Toiyama Y, Schetter AJ, Okugawa Y, Harris CC, Boland CR, Goel A (2015). Identification of a metastasis-specific MicroRNA signature in human colorectal cancer. *J Natl Cancer Inst*.

[42] Nguyen HV, Gore J, Zhong X, Savant SS, Deitz-McElyea S, Schmidt CM, House MG, Korc M (2016). MicroRNA Expression in a Readily Accessible Common Hepatic Artery Lymph Node Predicts Time to Pancreatic Cancer Recurrence Postresection. *J Gastrointest Surg*.

[43] Huang Z, Zhu D, Wu L, He M, Zhou X, Zhang L, Zhang H, Wang W, Zhu J, Cheng W, Chen Y, Fan Y, Qi L, Yin Y, Zhu W, Shu Y, Liu P (2017). Six Serum-Based miRNAs as Potential Diagnostic Biomarkers for Gastric Cancer. *Cancer Epidemiol Biomarkers Prev*.

[44] Yang S, He P, Wang J, Schetter A, Tang W, Funamizu N, Yanaga K, Uwagawa T, Satoskar AR, Gaedcke J, Bernhardt M, Ghadimi BM, Gaida MM, Bergmann F, Werner J, Ried T, Hanna N, Alexander HR, Hussain SP (2016). A Novel MIF Signaling Pathway Drives the Malignant Character of Pancreatic Cancer by Targeting NR3C2. *Cancer Res*.

[45] Gao Y, Xu Z, Yuan F, Li M (2018). Correlation of Expression Levels of Micro Ribonucleic Ccid-10b (miR-10b) and Micro Ribonucleic Acid-181b (miR-181b) with Gastric Cancer and Its Diagnostic Significance. *Med Sci Monit*.

[46] Xu C, Qi X (2020). MiR-10b inhibits migration and invasion of pancreatic ductal adenocarcinoma via regulating E2F7. *J Clin Lab Anal*.

[47] Peng Q, Shen Y, Lin K, Zou L, Shen Y, Zhu Y (2019). Identification of microRNA-92a and the related combination biomarkers as promising substrates in predicting risk, recurrence and poor survival of colorectal cancer. *J Cancer*.

[48] Peng Q, Feng Z, Shen Y, Zhu J, Zou L, Shen Y, Zhu Y (2019). Integrated analyses of microRNA-29 family and the related combination biomarkers demonstrate their widespread influence on risk, recurrence, metastasis and survival outcome in colorectal cancer. *Cancer Cell Int*.

[49] Icard P, Fournel L, Wu Z, Alifano M, Lincet H (2019). Interconnection between Metabolism and Cell Cycle in Cancer. *Trends Biochem Sci*.

[50] Espinoza-Sanchez NA, Gotte M (2020). Role of cell surface proteoglycans in cancer immunotherapy. *Semin Cancer Biol*.

[51] Montal ED, Dewi R, Bhalla K, Ou L, Hwang BJ, Ropell AE, Gordon C, Liu WJ, DeBerardinis RJ, Sudderth J, Twaddel W, Boros LG, Shroyer KR, Duraisamy S, Drapkin R, Powers RS, Rohde JM, Boxer MB, Wong KK, Girnun GD (2015). PEPCK Coordinates the Regulation of Central Carbon Metabolism to Promote Cancer Cell Growth. *Mol Cell*.

[52] Ma J, Matkar S, He X, Hua X (2018). FOXO family in regulating cancer and metabolism. *Semin Cancer Biol*.

[53] Anbarasan T, Bourdon JC (2019). The Emerging Landscape of p53 Isoforms in Physiology, Cancer and Degenerative Diseases. *Int J Mol Sci*.

[54] Meurette O, Mehlen P (2018). Notch Signaling in the Tumor Microenvironment. *Cancer Cell*.

[55] Guo J, Li P, Liu X, Li Y (2019). NOTCH signaling pathway and non-coding RNAs in cancer. *Pathol Res Pract*.

[56] Nagao A, Kobayashi M, Koyasu S, Chow CCT, Harada H (2019). HIF-1-Dependent Reprogramming of Glucose Metabolic Pathway of Cancer Cells and Its Therapeutic Significance. *Int J Mol Sci*.

[57] Soni S, Padwad YS (2017). HIF-1 in cancer therapy: two decade long story of a transcription factor. *Acta Oncol*.

[58] Zhou J, Yi Q, Tang L (2019). The roles of nuclear focal adhesion kinase (FAK) on Cancer: a focused review. *J Exp Clin Cancer Res*.

[59] Salminen A, Kauppinen A, Kaarniranta K (2019). AMPK activation inhibits the functions of myeloid-derived suppressor cells (MDSC): impact on cancer and aging. *J Mol Med (Berl)*.

[60] Peng Q, Yao W, Yu C, Zou L, Shen Y, Zhu Y, Cheng M, Feng Z, Xu B (2019). Identification of microRNA-181 as a promising biomarker for predicting the poor survival in colorectal cancer. *Cancer Med*.

[61] Ismail NI, Othman I, Abas F, N HL, Naidu R (2019). Mechanism of Apoptosis Induced by Curcumin in Colorectal Cancer. *Int J Mol Sci*.

[62] Alzahrani AS (2019). PI3K/Akt/mTOR inhibitors in cancer: At the bench and bedside. *Semin Cancer Biol*.

[63] Williams C, DiLeo A, Niv Y, Gustafsson JA (2016). Estrogen receptor beta as target for colorectal cancer prevention. *Cancer Lett*.

[64] Annibaldi A, Meier P (2018). Checkpoints in TNF-Induced Cell Death: Implications in Inflammation and Cancer. *Trends Mol Med*.

[65] Samaha D, Hamdo HH, Wilde M, Prause K, Arenz C (2019). Sphingolipid-Transporting Proteins as Cancer Therapeutic Targets. *Int J Mol Sci*.

[66] Griffin N, Faulkner S, Jobling P, Hondermarck H (2018). Targeting neurotrophin signaling in cancer: The renaissance. *Pharmacol Res*.

[67] Murugan AK (2019). mTOR: Role in cancer, metastasis and drug resistance. *Semin Cancer Biol*.

[68] Magaway C, Kim E, Jacinto E (2019). Targeting mTOR and Metabolism in Cancer: Lessons and Innovations. *Cells*.

[69] Mei L, Lu Z, Shen Z, Xu S (2020). The prognostic and diagnostic values of MicroRNA-10b in gastric cancer: A comprehensive study based on meta-analysis and TCGA database. *Medicine (Baltimore)*.

[70] Lu Y, Yao J, Yu J, Wei Q, Cao X (2014). The association between abnormal microRNA-10b expression and cancer risk: a meta-analysis. *Sci Rep*.

[71] Huang Q, Song Q, Zhong W, Chen Y, Liang L (2017). MicroRNA-10b and the clinical outcomes of various cancers: A systematic review and meta-analysis. *Clin Chim Acta*.

[72] Chen XL, Hong LL, Wang KL, Liu X, Wang JL, Lei L, Xu ZY, Cheng XD, Ling ZQ (2019). Deregulation of CSMD1 targeted by microRNA-10b drives gastric cancer progression through the NF-kappaB pathway. *Int J Biol Sci*.

